# Multiplicative processing in the modeling of cognitive activities in large neural networks

**DOI:** 10.1007/s12551-023-01074-5

**Published:** 2023-06-22

**Authors:** Juan C. Valle-Lisboa, Andrés Pomi, Eduardo Mizraji

**Affiliations:** 1 Group of Cognitive Systems Modeling, Biophysics and Systems Biology Section, Facultad de Ciencias, Universidad de la República, Iguá 4225, 11400 Montevideo, Uruguay; 2 Centro Interdisciplinario en Cognición para la Enseñanza y el Aprendizaje (CICEA), Universidad de la República, Espacio Interdisciplinario, 11200 Montevideo, Uruguay

**Keywords:** Multiplication, Tensor product, Context-dependent memory, Associative memories, Neural networks

## Abstract

Explaining the foundation of cognitive abilities in the processing of information by neural systems has been in the beginnings of biophysics since McCulloch and Pitts pioneered work within the biophysics school of Chicago in the 1940s and the interdisciplinary cybernetists meetings in the 1950s, inseparable from the birth of computing and artificial intelligence. Since then, neural network models have traveled a long path, both in the biophysical and the computational disciplines. The biological, neurocomputational aspect reached its representational maturity with the Distributed Associative Memory models developed in the early 70 s. In this framework, the inclusion of signal-signal multiplication within neural network models was presented as a necessity to provide matrix associative memories with adaptive, context-sensitive associations, while greatly enhancing their computational capabilities. In this review, we show that several of the most successful neural network models use a form of multiplication of signals. We present several classical models that included such kind of multiplication and the computational reasons for the inclusion. We then turn to the different proposals about the possible biophysical implementation that underlies these computational capacities. We pinpoint the important ideas put forth by different theoretical models using a tensor product representation and show that these models endow memories with the context-dependent adaptive capabilities necessary to allow for evolutionary adaptation to changing and unpredictable environments. Finally, we show how the powerful abilities of contemporary computationally deep-learning models, inspired in neural networks, also depend on multiplications, and discuss some perspectives in view of the wide panorama unfolded. The computational relevance of multiplications calls for the development of new avenues of research that uncover the mechanisms our nervous system uses to achieve multiplication.

## Introduction

Neural network models with the ability to process signals multiplicatively are a stage of neurocomputational network theory that began to develop in the 1970s. These multiplicative models were a sequel to the remarkable associative memory matrix models developed primarily in the early 1970s. These memory matrix models sought to explain the reliability of data storage in the face of partial deterioration of neural support, a fact long established by clinical neurology and by experimentation in animal models.

Matrix memory models were stimulated in the late 1960s by various suggestions, notably Gabor (1968), regarding the possibility that neural systems could support distributed data recording and storage, mathematically (not physically) analogous to the holograms of optics. Several authors independently contributed to the development of these matrix models, especially Anderson (1972) and Kohonen (1972).

But it was soon realized that along with their remarkable properties and their explanatory power, these matrix models had severe problems. In particular, they were not apt to branch their associations when the same key pattern was contextualized by different patterns. For example, when faced with the image of a dog, these memories had theoretical limits for associating that image with the different names that this animal has in different languages (e.g., the image of the dog associated with the contexts “English language” or “Spanish language” should be able to generate two divergent responses: “dog” and “perro” respectively). As a way of solving this problem and not losing the mathematical potential of matrix representations, since the mid-1970s, in particular due to the contributions of Poggio (1975) and Kohonen (1977), multiplicative models have been introduced. Varieties of these multiplicative models were developed and had an important expansion in the following years.

In this review, we cover more than fifty years of approaches that call for the inclusion of multiplicative processes in neural networks. In particular, we show how multiplication is used in these computational models. We also present the available evidence for how multiplication is carried on in biological networks. We review this domain of research including contributions that use networks with multiplicative processing from various angles. This is not intended to be an exhaustive review. We will focus on topics that we consider relevant for modeling cognitive functions and neuromimetic systems.

In the followign section, we will outline the nature of distributed memory models and their limitations. After that, we will analyze the theoretical and experimental arguments that have been developed to explain the appearance of multiplicative events in neural interactions. Then, we will show the various tensor representation formats proposed during the 1980s and their current developments. In the following section, we will show how these multiplicative processes are influencing powerful computational algorithms that are at the roots of modern artificial intelligence. Finally, we will present a perspective on the role of multiplicative models in neural computation.

## Distributed memory models

After the beginning of the mathematical theory of neural networks, with the binary neuron model of McCulloch and Pits (1943), and the random networks of Rapoport (1948), attention began to focus on modeling memory and learning. An important leap in research occurred when Frank Rosenblatt introduced a model, which he called the “Perceptron”, which was made up of a layer of interconnected formal neurons; this layer could be trained to identify patterns using a learning algorithm (Rosenblatt 1958). Rosenblatt’s learning algorithm involved changes in the strength of the connections between formal neurons (weights), which were a symbolic version of biological synapses. This algorithm was inspired by Hebb's idea of synaptic reinforcement as the basis of memories (Rosenblatt 1958). The model showed many potentialities, which were explored during the 1960s, but it also had important limitations. These limitations were especially emphasized in the detailed mathematical analysis of the model carried out by Minsky and Papert and published in their book "Perceptrons" (1969). One of the serious problems was the impossibility of training the Perceptron to distinguish patterns that were not linearly separable (Minsky and Papert 1969). A terse version of this inability was the impossibility of training by means of the Perceptron algorithm the logical operation Exclusive-OR (XOR). Based on this finding, the XOR became a kind of minimal test through which to evaluate the computational potentialities of a neural model associated with a learning algorithm.

At the same time, another fundamental problem of neural theory began to be analyzed through physical models of neural function: The problem of the reliability of neural memories when partial deterioration of their physical support occurred. This problem has been an unresolved enigma, born primarily from the experience of neurology that showed how, in certain fortunate cases, brain damage with significant loss of neuronal material (for example, caused by strokes or trauma) resulted in good preservation of the consolidated memories. In this way, several researchers suggested the possibility of finding neural models that, in their own physical logic of operation (action potentials, synapses, neurotransmitters, etc.), support a form of distributed data storage similar to optical holography (Longuet-Higgins 1968; Gabor 1968; Borsellino & Poggio 1972; Poggio 1973).

These suggestions stimulated the development of distributed memory models. To introduce them, let us begin by mentioning a remarkable model that links the biophysics and neurochemistry of synaptic transmission with the transduction of neuronal inputs to its output.

### The neuron model of Nass and Cooper

In this model, developed by Nass and Cooper (1975), the neuronal activity is assumed to be based on the modulation of the frequency of action potentials. A simplified version of this model is given by the following equation:

1 $$\mathrm{r}\left(\mathrm{i},\mathrm{t}+1\right)=\hspace{0.33em}\mathrm{H}\hspace{0.33em}\left[{\sum }_{\mathrm{j}=1}^{\mathrm{R}}{\mathrm{M}}_{\mathrm{ij}}.\mathrm{s}\left(\mathrm{j},\mathrm{t}\right)-\mathrm{U}\left(\mathrm{i}\right)\right]\hspace{0.33em}{\sum }_{\mathrm{j}=1}^{\mathrm{R}}{\mathrm{M}}_{\mathrm{ij}}.\mathrm{s}\left(\mathrm{j},\mathrm{t}\right)$$

where ${\mathrm{M}}_{\mathrm{ij}}$ is the weight of the synapse connecting axons j to neuron i, $\mathrm{U}\left(\mathrm{i}\right)$ is specific the threshold of neuron i, $\mathrm{s}\left(\mathrm{j},\mathrm{t}\right)$ is the frequency of action potentials coming via axon j, and $\mathrm{r}\left(\mathrm{i},\mathrm{t}+1\right)$ is the output of neuron i in the following time step, and H is the Heaviside function ($\mathrm{H}(\mathrm{x})=0$ if $\mathrm{x}\le 0$ and $\mathrm{H}(\mathrm{x})=1$ if $\mathrm{x}>0$). A basic hypothesis is that this neuron integrates a large neural network. Under this hypothesis, each neuron receives thousands of inputs that generate a basal noise ${\mathrm{s}}_{0}\left(\mathrm{j}\right)$ that produces a basal output ${\mathrm{r}}_{0}\left(\mathrm{i}\right)$. Assuming that the abundance of inputs represented by ${\sum }_{\mathrm{j}=1}^{\mathrm{R}}{\mathrm{M}}_{\mathrm{ij}}.\mathrm{s}\left(\mathrm{j},\mathrm{t}\right)$ pushes the activity of the neuron beyond the threshold U(i), then the neuron activity occurs inside the linear region of Eq. (1).

Now, we show how to simplify the output-inputs relation of model (2) redefining the inputs and output as follows:

$$\begin{array}{cc}\mathrm{f}\left(\mathrm{j}\right)=\mathrm{s}\left(\mathrm{j}\right)-{\mathrm{s}}_{0}\left(\mathrm{j}\right)& \mathrm{g}\left(\mathrm{i}\right)=\mathrm{r}\left(\mathrm{i}\right)-{\mathrm{r}}_{0}\left(\mathrm{i}\right)\end{array}$$

Hence, in the region near the basal states the output $g(i)$ is approximate by a linear combination of the inputs $\mathrm{f}(\mathrm{j})$:

2 $$\mathrm{g}(\mathrm{i},\mathrm{t}+1)={\sum }_{\mathrm{j}=1}^{\mathrm{R}}{\mathrm{M}}_{\mathrm{ij}}\mathrm{f}(\mathrm{j},\mathrm{t})$$

These variables $\mathrm{g}(\mathrm{i})$ and $\mathrm{f}(\mathrm{j})$ measure the deviations of the spike frequencies from their basal values. Consequently, they are positive, null, or negative real numbers.

A group of Q neurons subjected to R inputs can be described by the following matrix equation:

3 $$\mathrm{g}=\mathrm{Mf}$$

where f and g are column vectors

$$\begin{array}{cc}\mathrm{f}=[\mathrm{f}(1)\mathrm{f}(2)\cdots \mathrm{f}(\mathrm{R}){]}^{\mathrm{T}}& \mathrm{g}=[\mathrm{g}(1)\mathrm{g}(2)\cdots \mathrm{g}(\mathrm{Q}){]}^{\mathrm{T}}\end{array}$$

The components of matrix are the synaptic coefficients:

$$\mathrm{M}=[{\mathrm{M}}_{\mathrm{ij}}]\in {\mathbb{R}}^{\mathrm{R}\times \mathrm{Q}}$$

These models generated two innovative approaches. First, they assumed that the basic neural code was not necessarily an isolated action potential (and therefore a binary signal) but could encompass a continuous code over a certain interval (e.g., spike frequency). Second, they introduced neural vectors as the basic units of nervous system activity. In this framework, information on neural patterns was represented by temporally variable signals carried by sets of thousands or tens of thousands of neurons in parallel. The original model of Nass and Cooper (1975) (not our simplified version shown in Eq. (1)) has the virtue of being a model that incorporates biophysical data of the membranes and synaptic neurotransmission. But nearly identical mathematical behavior can be derived from electrical circuit models of neuronal function (see Kohonen 1977, page 137).

### Distributed associative memories

These models were developed (in many cases independently) by many authors: Anderson (1972), Kohonen (1972), Cooper (1974), and Amari (1977a, b), among others. In this vector–matrix format, an associative memory Mem can be defined as a set of K pairs of associated output-input vectors: $\mathrm{Mem}=\left\{\left({\mathrm{g}}_{\mathrm{k}},{\mathrm{f}}_{\mathrm{k}}\right):\mathrm{i}=1,\cdots ,\mathrm{K}\right\}$. In an ideal situation, in the presence of an input vector ${\mathrm{f}}_{\mathrm{k}}$ belonging to the set of associated pairs, the output of the memory will be exactly ${\mathrm{g}}_{\mathrm{k}}={\mathrm{Mf}}_{\mathrm{k}}$. The difficult problem is to find the matrix M that implements a memory Mem. Approximate optimal solutions to this problem were obtained by Kohonen using pseudoinverse matrices (Kohonen 1977). However, if we assume that the inputs are orthonormal, an elegant and minimalist (because many realistic aspects are deliberately omitted) exact solution emerges:

4 $$\mathrm{M}={\sum }_{\mathrm{i}=1}^{\mathrm{K}}{\mathrm{g}}_{\mathrm{i}}{\mathrm{f}}_{\mathrm{i}}^{\mathrm{T}}$$

(superscript T means transposition). This matrix shows clearly the operating way of this memory. Processing the input ${\mathrm{f}}_{\mathrm{k}}$ the matrix (4) produces

5 $${\mathrm{Mf}}_{\mathrm{k}}={\sum }_{\mathrm{i}=1}^{\mathrm{K}}{\mathrm{g}}_{\mathrm{i}}\langle {\mathrm{f}}_{\mathrm{i}},{\mathrm{f}}_{\mathrm{k}}\rangle ={\mathrm{g}}_{\mathrm{k}}$$

The inner products $\langle {\mathrm{f}}_{\mathrm{i}},{\mathrm{f}}_{\mathrm{k}}\rangle$ act as filters: in the case illustrated in Eq. (5), one of them $\langle {\mathrm{f}}_{\mathrm{k}},{\mathrm{f}}_{\mathrm{k}}\rangle =1$ and the others are zero. They are all zero if the input is not in the memory. The fundamental holographic-like property of these matrix memories is evident if we analyze the structure of the coefficients ${\mathrm{M}}_{\mathrm{\alpha \beta }}$ of matrix (4):

6 $${\mathrm{M}}_{\mathrm{\alpha \beta }}={\sum }_{\mathrm{i}=1}^{\mathrm{K}}{\mathrm{g}}_{\mathrm{i}}(\mathrm{\alpha }){\mathrm{f}}_{\mathrm{i}}(\upbeta )$$

This remarkable equation shows (a) that components of all the vectors pairs of the memory are scattered through the matrix coefficients, and (b) that data are superimposed on each synaptic coefficient. Point (a) gives an explanation for reliability: if the matrix memory is very large, the destruction of some synapses is not enough to delete the information, eventually producing a slight data corruption. Point (b) shows how data coming from different associated pairs are subtly incorporated into the same material support (e.g., synaptic molecular receptors).

We illustrate the distributed and superimposed nature of the memory with a miniature example. Let $\mathrm {{f}_{1}={\left[a b c\right]}^{T}}$ and $\mathrm{ {f}_{2}={\left[d e f\right]}^{T}}$ be the inputs of a matrix memory, and $\mathrm {{g}_{1}={\left[\alpha \beta \right]}^{T}}$ and $\mathrm {{g}_{2}={\left[\gamma \delta \right]}^{T}}$ their associated outputs. Note that all four vectors are column vectors (annotated using transposition). Our miniature memory is defined by

$$\mathrm {M={g}_{1}{{ f}_{1}}^{T}+{g}_{2} {{f}_{2}}^{T}}$$

The inner structure of M is

$$\mathrm {M=\left[\begin{array}{ccc}{\mathrm m}_{11}& {\mathrm m}_{12}& {\tt \mathrm m}_{13}\\ {\mathrm m}_{21}& {\mathrm m}_{22}& {\mathrm m}_{23}\end{array}\right]=\left[\begin{array}{ccc}\mathrm \alpha a+ \gamma \mathrm d& \alpha \mathrm b+\gamma \mathrm e& \alpha \mathrm c+\gamma \mathrm f\\ \beta \mathrm a+ \delta \mathrm d& \beta \mathrm b+\delta \mathrm e& \beta \mathrm c+\delta \mathrm f\end{array}\right]}$$

The scattering of data is seen in the fact that the components of each input (and output) vector are spread over the different coefficients of the matrix. The superimposition is seen in the fact that a matrix coefficient includes the addition of components belonging to different pairs of input and output vectors.

In these memories, the number of associated pairs is limited by memory capacity. It is assumed that for a memory module, the inputs all have the same dimension, and the same is assumed for the outputs, whose dimension is in general different from that of the inputs. These dimensions are dictated by the anatomical connectivity of the memory modules. A corollary of this neural vector representation of patterns was that associative memories were mapped onto large-dimensional matrices.

These matrix associative memories can acquire new data through various supervised training procedures. A very powerful one arises from the method presented by Widrow and Hoff (1960), which is completely adaptable to the vector format of the inputs and outputs. It is a gradient-descending procedure that seeks to minimize the error between the output to be trained and the successive outputs produced by memory. At the end of the process, an output close to the one sought is obtained, and the structure of the matrix is globally modified. This Widrow-Hoff procedure is a refinement of Hebb’s idea of learning through synaptic consolidation. Another alternative procedure to incorporate new information into memory was developed by Kohonen (1977) using Greville’s theorem on pseudoinverse matrices. These two training methods can continue to incorporate associated pairs of vectors into memory until a critical level of Signal to Noise ratio is reached, after which interference no longer allows good discrimination.

### A shortcoming of these models: their impossibility of adaptive associations

**Fig. 1:**
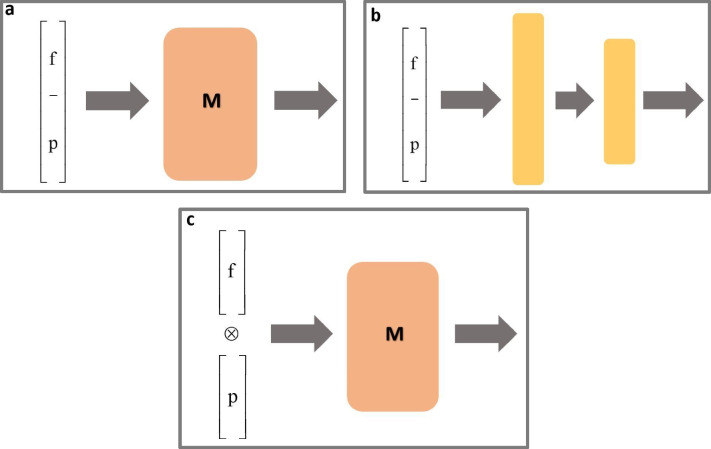
Proposals for adaptive (context-dependent) associations. **a** Additive contextualization by enlarging the input vector f with components representing context p. M is a one single layer memory matrix. Left arrow represents connectivity from each component of the entry to each unit of the memory layer. **b** Multilayer perceptron: the additive enlargement of input vector f with context units p is processed by two successive hidden layers. Neuronal units of successive layers are fully connected. **c** The tensor product ( ⊗) of input vector f with context vector p is processed by a single layer memory matrix

Now we show a weakness of these pioneering models of matrix memories that appears when trying to incorporate the notion of context. Any pattern that will be identified or associated by a biological memory is always submerged in a larger environment of neural activity that can be considered as its context. Whatever the type of neural activity that is acting as context (sensory impressions or cognitive information), it can be represented by means of vectors that encode that information (for a general approach to the integration of patterns see Morrison et al. 2001). Let us imagine that these memories are faced with a pattern (let’s call it a “key pattern”) accompanied by two vector contexts with the same dimension between them and that adds this dimension to that of the key pattern (consequently, the dimension of this vector is the sum of that of the key pattern and that of the context). It happens that under these conditions, memory is not capable of orienting its associations towards two different and eventually arbitrary outputs. Let’s take as a simple example the problem of associating an object, (e.g., a book) with the name of that object in two different languages (e.g., “book” or “libro”). A matrix memory cannot generally be trained for these uncorrelated names, unlike a biological memory, which has full capacity to allow an object to be associated with an arbitrary name or a neologism (it is quite possible to create a new and arbitrary name for the book object, e.g., "libuk"). This incapacity of matrix memories has been formally proven by Hinton (1989); a condensed proof can be seen in Mizraji et al. (1994, p. 148). These proofs show that matrix memories share with perceptrons the inability to distinguish linearly separable patterns, which leads again to the XOR problem (Fig. 1).

The most widely used procedure to solve this problem is the design of neural models with hidden layers between the layer that received the input and the one that generated the output. This procedure creates a class of extremely relevant neural models. In these models, the synapses are adjusted by a powerful algorithm usually called “backpropagation”, which adjusts synaptic weights by a descending gradient algorithm. This algorithm was discovered several times by various independent authors, but widely disseminated through the articles by Rumelhart and McClelland and the “Parallel Distributed Processing” (PDP) Group (Rumelhart, Hinton and Williams 1986a, b; Werbos 1994). Backpropagation can also be used to train linear networks with several “hidden” layers (Saxe, McClelland, and Ganguli, 2013) but the final mapping learned in such a network is no more powerful than a single layer (as the product of several matrices is a matrix). This algorithm is particularly useful when there are non-linear links between the formal neurons of some of its layers. The use of nonlinear activity functions distorts the matrix representation for the memories, but the representation of information by means of neural vectors and the distributed storage of data in the synapses of the model are retained. These hidden layer networks solve the context problem and therefore the XOR problem (which became a test of the computational power of neural networks), at the expense of obscuring their inner workings. In this way, the theoretical power of the matrix representation was replaced by the computational power of the nonlinear model.

The other procedure to provide these models with the capacity that the same pattern of neural activity acting as an input can be associated with different output patterns of neural activity depending on the context, was proposed at the end of the 80 s and will be the subject of Sect. 4. These models, based on a tensor composition of the inputs to memory, have the great advantage that they preserve the matrix representation, enabling the development of the theory, without being condemned to try variations of computational algorithms that improve performance, but acting like black boxes. The tensor composition of the inputs requires the multiplication of neural signals. Before presenting this second possibility of making context-dependent associations, we will refer in the following Section to the presence of multiplications in previous models of neural networks.

## Multiplications in early neural models and in biological neurons

Multiplication is the simplest form of a non-linearity and was early proposed as a form of increasing the computational power of neural models (Koch and Poggio 1992). The presence of multiplication in real neurons was detected in several sensory processing systems, as the localization of sound (Peña and Konishi 2001), the combination of multisensory signals (Huston and Krapp 2009), and the computation of visual motion (Hassenstein and Reichardt 1956). More evidence has been found in the context of binocular interaction (Freeman 2004), attentional modulation (Treue and Trujillo 1999; McAdams and Maunsell 2000), and motor planning (Hwang et al 2003).

The possibility of multiplication of signals in neurons has been explored via biophysical models, computer simulations and experimental data. A model neuron including the possibility of multiplicative effects among signals can be written as follows:

7 $$\mathrm{g}(\mathrm{i},\mathrm{t}+1)={\sum }_{\mathrm{j}}{\mathrm{M}}_{\mathrm{ij}}^{(1)}\mathrm{p}(\mathrm{j},\mathrm{t})+{\sum }_{\mathrm{k}}{\mathrm{M}}_{\mathrm{ik}}^{(2)}\mathrm{f}(\mathrm{k},\mathrm{t})+{\sum }_{\mathrm{j},\mathrm{k}}{\mathrm{M}}_{\mathrm{i}(\mathrm{jk})}^{(3)}\mathrm{p}(\mathrm{j},\mathrm{t})\mathrm{f}(\mathrm{k},\mathrm{t})$$

The first two sums represent signals coming from two separate groups of neurons, e.g., one codifying a context and another codifying a sensory input, and the third double sum represents the effect of multiplicative interactions among these signals. Consequently, different conditions modulating synaptic strengths lead to different magnitudes of the synaptic coefficients ${\mathrm{M}}^{(\uplambda )},\uplambda =1..3.$

The biophysical mechanisms responsible for these multiplicative behaviors in biological neurons remain a topic of debate and research. Let us previously mention that it has been shown that multiplicative responses can arise in a network through population effects, with neurons that do not perform multiplication of signals individually. “A recurrently connected network with excitatory connections between similarly tuned neurons and inhibitory connections between differently tuned neurons can perform a product operation on additive synaptic inputs” (Salinas and Abbott 1996).

It is important to note that the general multiplicative framework of Eq. (7) has also appeared in recent developments in the rich and traditional area of Neural Field Theory, an approach in which the interaction of billions of neurons is treated as a continuum (Coombes et al. 2014). The advances provided by the renewed conception of *transient brain dynamics*, considering the temporal evolution of mental life as sequences and transient interactions of metastable states (Rabinovich et al 2008), together with the constructive theory of *dynamic cognitive models* (beim Graben and Potthast 2009), have endowed these models with the capacity to present the type of adaptive dynamics necessary to model cognitive activity. In particular, beim Graben and Potthast (2012) and beim Graben and Hutt (2014), building from the Amari equation for a neural field (Amari 1977a, b), where synaptic weight matrices are represented by integral kernels and the product between a matrix and a vector of neural activity becomes approximated by an integral over a synaptic kernel and a neural field, perform the expansion of this integral in the presence of a nonlinear activation function into a Volterra series, obtaining an expression with Eq. (7) describing the two main terms. This provides another possibility for the biological realization of multiplication, different from the lineage of discrete-time models that we discuss in what follows.

Now we review some of the neural network models that have included multiplications and several of the biophysical mechanisms proposed to perform multiplicative effects.

### Multiplications in signal processing

Although the focus of our interest will be the multiplication of two neural signals that converge on a same neural unit, it is convenient to be aware beforehand that the processing of a signal through the multiplication operation is present, inevitably, from the first formal models in neurosciences. Indeed, since McCulloch-Pitts’ first model, the effect of a nerve signal on a neuron is capable of being multiplicatively modulated by a synaptic weight, capable of increasing or decreasing the relative importance or strength of that input, modifying the gain of the signal.

In the original paper, this multiplicative effect was achieved not by varying the strength of a synaptic contact, but by adding synaptic endings from the same axon. As in the origin of the arithmetic operation: to multiply is to add an integer number of times. This original idea was later refined and reinterpreted as a single synaptic weight that modifies the gain of a neural input by multiplying it by a real number.

This subtlety in interpreting a multiplication is important, in fact, it provides two different ways to achieve a multiplicative effect: by adding or cloning similar elements, or by generating a subcellular “environment” that enhances the efficiency of an input, and these possibilities must be carried out at the level of the synapse itself (in one or more of its participating cellular or molecular elements) or at the level of the dendrites (through structural, molecular variations or the subcellular environment of chemical mediators). Let us note then that the multiplicative effect of a second input can be seen as a change in synaptic weight that affects the first through a change in the cellular environment that must process it.

A ‘multiplicative’ effect over an afferent neural signal, either produced by the action of a synaptic weight or by another signal coming from a different afferent neuron, may use the same cellular and molecular available mechanisms.

### Logical multiplication and coincidence detectors

In the models in which the activity of the neurons is represented by a binary variable, the only possible multiplication of the inputs is the logical multiplication, through the implementation of the AND function. The logical conjunction, in effect, shares with the more general multiplication of two real numbers, the property that if one of the entries has no activity (has value 0), there is no response. In other words, for a neuron to discharge, it is necessary the temporal coincidence of the activity of its afferences. A “coincidence detector”, then, behaves like a mechanism that can gate the flow of information.

Neurons in nervous systems have been reported to respond preferentially to synchronized synaptic inputs (König et al. 1996; Agmon-Snir et al. 1998; Joris et al. 1998) and theoretical results also have emphasized the role of the synchronous firing of neurons for information processing in the brain and the information carried by single spikes (Bialek and Zee 1990).

Srinivasan and Bernard (1976) showed that if neurons detect coincident arrivals of spikes from two input neurons, they can function as multipliers of the average spike frequency of their inputs.

### Multiplicative effects with integrate and fire neurons

The leaky integrate and fire (LIF) model (Stein 1967) has been used to model physiologically realistic spike trains. Bugmann (1991, 1992) demonstrated the existence of a multiplicative regime for a LIF neuron based on a coincidence detector operation.

A logarithmic stimulus–response relation has been observed in real neurons since the 60 s (Ratliff 1965) and also postulated in neural modeling literature (Koch and Poggio 1992; Yeshurun and Schwartz 1989). Tal and Schwartz (1997) provide a biophysical mechanism to perform this logarithmic transfer function. They show that in leaky integrate and fire neurons, a broad range of the ratio of refractory period duration to membrane time-constant yields a logarithmic transfer function. Then, LIF neurons can be used to multiply neural signals by addition of two LIF neuron outputs, yielding the logarithm of the product.

### The Sigma-pi neuron and “functional-link nets”

Sigma-pi neurons were initially proposed by Feldman and Ballard (1982), and then by the influential PDP group in the mid-1980s (Rumelhart, Hinton and McClelland 1986) as part of the toolbox of the new neural modeling paradigm. A sigma-pi neuron (i) has its entries partitioned in different pools. The activities of all the K neurons in a pool (j) are multiplied. Then the neuron performs a weighted sum of these products that come from the different pools:

8 $${\mathrm{a}}_{\mathrm{i}}={\sum }_{\mathrm{j}}{\mathrm{w}}_{\text{ij}}{\prod }_{\mathrm{k}}{\mathrm{a}}_{\text{j1}}{\mathrm{a}}_{\text{j2}}...{\mathrm{a}}_{\text{jK}}$$

Williams (1986), stated that for all practical purposes, no more than two neurons were needed in each multiplicative pool in the models used.

Valle-Lisboa et al (2005) showed that the context-dependent associators that perform the tensor product of their vector inputs (see Sect. 4) can be seen to be composed of a particularly convenient special case of sigma-pi neurons that admit a powerful algebraic representation.

“Functional-link net” is a system architecture and a network computational approach developed with the goal of devising a general-purpose artificial neural-net computer (Pao 1989; Pao and Takefuji 1992). Using this category of nonlinear mappings, Pao (1989) compared the XOR training speed for a network whose inputs were neural vectors with products of their components (trained by the Widrow-Hoff algorithm), versus a network of hidden layers trained by backpropagation. Pao found that the network with nonlinear vectors acquired the XOR with much fewer training steps than the network with hidden layers: a suggestive result that encouraged the search for models that included forms of non-linear processing as alternatives to solve contextualization problems.

### Neurobiological mechanisms postulated to perform multiplications in a neuron

In addition to the mechanisms mentioned in the models of LIF neurons, various other proposals have been made to multiply the signals that reach a neuron. Biophysical mechanism implied in neural multiplication have been reviewed in the classical works of Koch and Poggio (1992), Koch (1999), Koch and Segev (2000) and Silver (2010) among others.

Among the many mechanisms proposed, we want to highlight those that rest on dendritic processing. Dendrites with their spines, the capacity of clustering of the synaptic inputs and the variety of passive and active responses, have been proved to generate nonlinear interactions in the processing of neural afferences to a neuron (Koch et al. 1983; Mel 1993; London and Häusser 2005).

Of particular relevance for this review is the role of NMDA receptor in coincidence detection and in Hebbian associative learning (Seeburg et al 1995; Yuste et al 1999; Tabone and Ramaswami 2012).

Recently, a new mechanism has been envisioned: a ‘multiplicative disinhibition’ arising from the coincidence of excitation and release from shunting inhibition (Groschner et al 2022).

### Nonlinearities in matrix models of associative memory

As early as the 1970s, the limitations of the linear approach for distributed associative memories were well known. In 1975, Poggio analyzed what he called “Optimal nonlinear associative recall” (Poggio 1975) a general framework for determining the nonlinear function which optimally associates (on given criteria) two sets of data given by discrete, finite column vectors forming two matrices X (“input”) and Y (“output”) with the same numbers of columns and an arbitrary numbers of rows. The optimal solution in least squares sense is a polynomial mapping of degree k on X. In this analysis, the matrix structure of distributed memories is preserved. Poggio also provides an iterative method which was used by Kohonen to analyze these nonlinear maps and perform some numerical experiments (Kohonen 1977, p. 83).

In the next section, we will present a solution to the contextualization problem in associative networks based on the tensor product of the inputs of a matrix memory. But before, it should be noted that tensor models were previously used by Pellionisz and Llinás to propose a way of how the brain may implement functional geometries involved in sensory motor transformations (Pellionisz and Llinás 1979, 1985).

## Multiplicative contexts in matrix memories

During the 1980s, various models of distributed memory with tensor components were deployed. These models allowed expanding the computational capabilities of matrix memories and solving the problem of adapting the outputs to different contexts. The models we will mention had different mathematical formats and different motivations due to the varied backgrounds of the researchers. But they all converged to matrix or tensor structures that associated Outputs (O), Contexts (C) and Inputs (I). In these triplets (O, C, I) the elements were multidimensional objects, assimilable in all cases to neural vectors.

### Models with tensor product representation

Perhaps the pioneering work using multiplicative processing was published by Pike (1984), where the author develops an operation between matrices that produces a scalar product of their components. When matrices have a structure of outer products ${\mathrm{ab}}^{\mathrm{T}}$ and ${\mathrm{cd}}^{\mathrm{T}}$ the scalar generated corresponds to the product of two inner products (Pike 1984, p. 284). This operation was used by Humphreys et al. (1989), in an article where the varieties of memory modalities are analyzed from the perspective of cognitive psychology. These authors use matrix memories and describe three-dimensional arrays of vectors that manage to filter the inputs by means of two inner products (Humphreys et al. 1989, p. 215) their work shows a tensor neural model that illustrates the potentialities of the approach.

Dolan and Smolensky (1989), in a framework that brings together classical artificial intelligence and cognitive science, propose a tensor product between vectors that they call “roles” and others they call “fillers”. The objective of their article is to analyze the possibility that connectionist networks represent and process cognitive structures, in particular trees and structured representations. This article seeks to reconcile some traditional cognitive science theories with connectionist approaches (a topic of intense controversy and disagreement at the time). Although the focus of the article departs form the associative memory tradition, its mathematics uses the matrix formats typical of distributed memories, and there the tensor triples (O, C, I) are clearly expressed in terms of vectors. The filter by double inner products appears clearly in Dolan and Smolensky (1989, p. 58). Shortly thereafter, Smolensky (1990) published a lengthy paper using a rigorous mathematical formalism that expands on the theoretical approach he had published in his paper with Dolan.

Starting from a biophysical approach, Mizraji (1989) publishes an article where he raises the problem of contexts in matrix memories. The tensor model of this article is based on two fundamental biological constraints: (a) the need for neural memories to be adaptive systems in the sense of Ross Ashby (1958), so that associations can be modulated by vector contexts, and (b) that the principle of “gratuity” (Monod 1967) operates, so that contexts, inputs, and outputs do not have forced structural links to each other for example, that they are free to use an arbitrary name for a book (“libuk”) as was mentioned in Sect. 2. We mention in passing that the idea of “gratuity” was discovered by Jacques Monod in the context of molecular biology. In his classic book “Chance and Necessity”, he describes this notion as follows: “This fundamental concept of gratuity- i.e., the independence, chemically speaking, between the function itself and the nature of the chemical signals controlling it- applies to allosteric enzymes. In this case one and the same protein molecule does double duty as specific catalyst and as transducer of chemical signals” (Monod 1971). In the neural environment, gratuity implies the necessity for cognitive adaptive behavior of non-constrained links between the key inputs and their contexts. This work shows that one way of contextualizing while retaining the matrix structure of the memories, and subjecting the procedure to the mentioned constraints, is to perform a Kronecker product between the context and the key input, and associate this dual input with the different outputs. This is how, here too, the triplet (O, C, I) arises and the filtering by double internal products emerges immediately as a consequence of formalism (Mizraji 1989, p. 197).

If we have insisted so much on the double filter by inner products (in which the aforementioned works agree despite their different approaches), it is because therein lies the broad computational potential of these multiplicative models. This double filter is the key to encoding contextualized patterns without having to resort to hidden layers, and to be able to train the networks using the Widrow-Hoff algorithm, generally much simpler to execute than Backpropagation.

The search for the dynamics of cognitive processes has given rise to the natural appearance of tensor neural models. In beim Graben and Potthast (2009), the authors connect the abstract symbolic representations of cognitive processes, with their representation through vector spaces where tensor operations are installed and allow the generation of neural dynamic systems. In language analysis, tensor models have had an important presence. An investigation on the difficult problem of understanding how the grammatical structures of natural language are implemented in a physical support such as the human brain has been carried out by beim Graben and Gerth (2012). The authors show in a parsimonious way how the formalism of grammars, with their hierarchical structures and the ramifications of their trees, can move constructively from abstract representations to vectors and tensor products; this establishes the link with connectionist models, and consequently, with their potential neural implementation (beim Graben and Gerth 2012). In beim Graben el al (2022) vector symbolic architectures (VSA) and associated tensor representations are discussed in detail as a versatile way of representing a wide variety of grammatical structures. In their article, the authors accompany the formal theory with a computational procedure and update the link between connectionist neural models and artificial intelligence (beim Graben et al. 2022). Other VSA and hyperdimensional computing models create a binding conceptually equivalent to forming the tensor product, and this tensor product is projected to a lower dimensional vector space. The low dimensional vectors so obtained are an approximation to the fully accurate tensor representations, trading off mathematical precision for computational advantages. This idea was developed by Plate (1994), Gayler (1998) and Kanerva (2009).

### Context-dependent associative memories

Let us dedicate the rest of this section to the various derivations of the biophysical model described in Mizraji (1989). We are now going to present the contextualized memories following the simple format described that article.

The Kronecker product for arbitrary matrices $\mathrm{U}=[{\mathrm{u}}_{\mathrm{ij}}]\in {\mathbb{R}}^{\mathrm{m}\times \mathrm{n}}$ and $\mathrm{V}=[{\mathrm{v}}_{\mathrm{ij}}]\in {\mathbb{R}}^{\mathrm{p}\times \mathrm{q}}$ is defined as

$$\mathrm{U}\otimes \mathrm{V}=[{\mathrm{u}}_{\mathrm{ij}}\mathrm{V}]\in {\mathbb{R}}^{(\mathrm{mp})\times (\mathrm{nq})}$$

The basic properties of this product are:

$$(\mathrm{a})\uplambda \left(\mathrm{A}\otimes \mathrm{B}\right)=\mathrm{A}\otimes \left(\mathrm{\lambda B}\right)$$

$$(\mathrm{b}) (\mathrm{A}\otimes \mathrm{B}{)}^{\mathrm{T}}={\mathrm{A}}^{\mathrm{T}}\otimes {\mathrm{B}}^{\mathrm{T}}$$

$$(\mathrm{c})\mathrm{ A}\otimes \mathrm{B}+\mathrm{A}\otimes \mathrm{C}=\mathrm{A}\otimes \left(\mathrm{B}+\mathrm{C}\right)$$

$$(\mathrm{d})\left(\mathrm{A}\otimes \mathrm{B}\right)\left(\mathrm{C}\otimes \mathrm{D}\right)=\left(\mathrm{AC}\right)\otimes \left(\mathrm{BD}\right)$$

Here, A, B, C and D are arbitrary matrices (as long as they comply with the dimensional conformability of the operations), and $\uplambda$ is a scalar.

To describe context-dependent matrix memories, we will assume the simplest case, where all input and context vectors are orthonormal. These memories would exhibit the following structure

9 $$\mathrm{M}={\sum }_{\mathrm{i},\mathrm{j}}{\mathrm{g}}_{\mathrm{ij}}({\mathrm{p}}_{\mathrm{i}}\otimes {\mathrm{f}}_{\mathrm{j}}{)}^{\mathrm{T}}$$

where ${\mathrm{g}}_{\mathrm{ij}}$ represents the output associated to the input ${\mathrm{f}}_{\mathrm{j}}$ in the context ${\mathrm{p}}_{\mathrm{i}}$. Conseqently, an input ${\mathrm{f}}_{\mathrm{h}}$ in the context ${\mathrm{p}}_{\mathrm{k}}$ is processed as follows:

10 $$\mathrm{M}({\mathrm{p}}_{\mathrm{k}}\otimes {\mathrm{f}}_{\mathrm{h}})={\sum }_{\mathrm{i},\mathrm{j}}{\mathrm{g}}_{\mathrm{ij}}\langle {\mathrm{p}}_{\mathrm{i}},{\mathrm{p}}_{\mathrm{k}}\rangle \langle {\mathrm{f}}_{\mathrm{j}},{\mathrm{f}}_{\mathrm{h}}\rangle$$

Here we can clearly see the emergence of the double filter that this model creates, a consequence of property (d) of the Kronecker product.

Let us imagine, to illustrate its computational capacity, a minimalist contextualized memory, which describes how the visual input f associated with a book, can modify its output according to the required idiomatic context. (e.g., ${\mathrm{p}}_{1}$ ask for the name in English and ${\mathrm{p}}_{2}$ in Spanish). This small memory is

$$\mathrm{M}={\mathrm{g}}_{1}({\mathrm{p}}_{1}\otimes \mathrm{f}{)}^{\mathrm{T}}+{\mathrm{g}}_{2}({\mathrm{p}}_{2}\otimes \mathrm{f}{)}^{\mathrm{T}}$$

and if the context is ${\mathrm{p}}_{2}$ we get

$$\mathrm{M}({\mathrm{p}}_{2}\otimes \mathrm{f})={\mathrm{g}}_{1}\langle {\mathrm{p}}_{1},{\mathrm{p}}_{2}\rangle \langle \mathrm{f},\mathrm{f}\rangle +{\mathrm{g}}_{2}\langle {\mathrm{p}}_{2},{\mathrm{p}}_{2}\rangle \langle \mathrm{f},\mathrm{f}\rangle ={\mathrm{g}}_{2}$$

hence, “libro” (${\mathrm{g}}_{2}$). Note that due to orthonormality it is $\langle {\mathrm{p}}_{1},{\mathrm{p}}_{2}\rangle =0$ and $\langle {\mathrm{p}}_{2},{\mathrm{p}}_{2}\rangle =\langle \mathrm{f},\mathrm{f}\rangle =1$.

But for the biophysical approach in which this model was developed, it was clear that Kronecker’s product, a too perfect mathematical operation, could not exist in real neural structures. However, experiments of random removal of components from a memory matrix with the structure of the matrix given in Eq. (9) show a strong degree of tolerance to destruction, measured by the correlation between the current output vector, and the ideal trained output vector. An example of this is shown in Fig. 2 from Pomi and Mizraji (1999). In that article, the “ideal” Kronecker product is reinterpreted as a situation in which each component of the key input is weighted by all elements of the context vector, a fact that emerges immediately from the formal definition of the Kronecker product. In contrast, a real condition could be interpreted as a statistical Kronecker product, where each key input component is weighted by a statistical sample of the context vector components (Pomi and Mizraji 1999). Let us note that there is a symmetrical situation here and we could in the previous comment swap key vector for context vector, since in fact, the nullification by a weight = 0 of some components (of the context or of the key vector) deletes them both. Since the signal-to-noise ratio of matrix memories already established by the creators of the theory (Anderson 1972; Kohonen1972) increases with the size of the memory, the dimensional expansion that creates the Kronecker product enhances this ratio and gives more space for the disappearance of elements, nevertheless maintaining an acceptable quality of the associations.

**Fig. 2:**
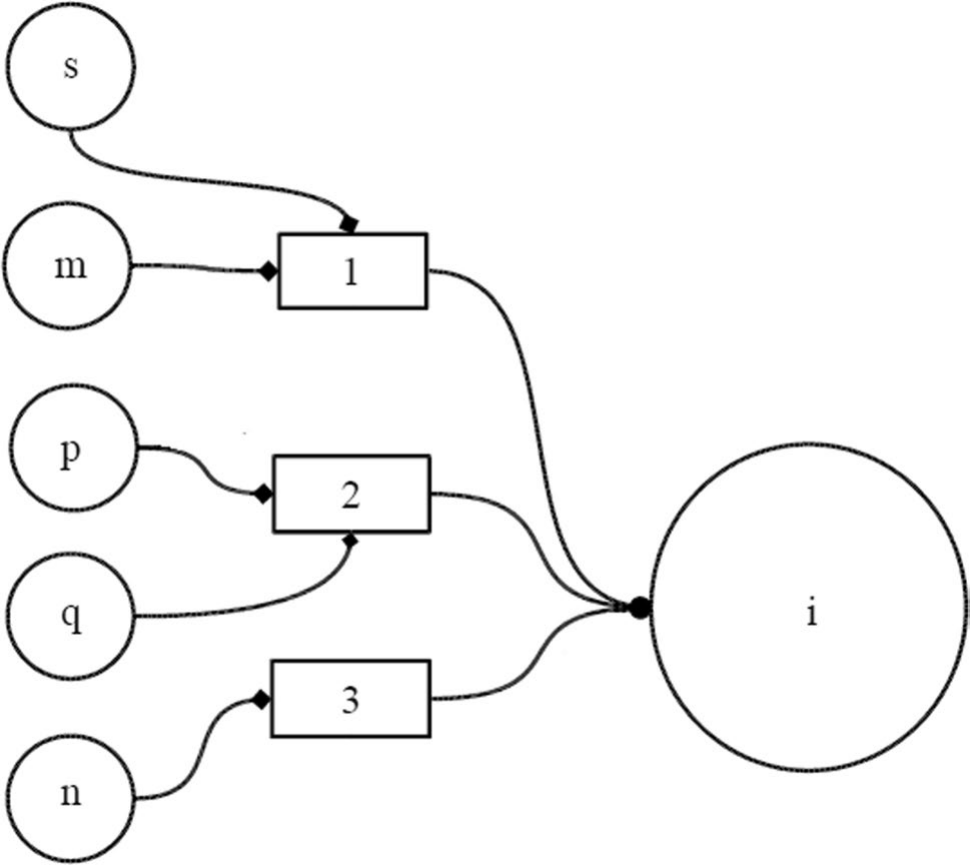
Diagram of a sigma-pi neuron. Input units (s, m, p, q, n) are partitioned in three disjoint sets. The activities of the neurons within each one of these pools are multiplied (blocks 1, 2, and 3). Neuron (i) performs a weighted sum of these products. Adapted from Valle-Lisboa et al. (2005)

### Symbolic and computational potentialities

We want to make two comments about the potential relationships of this relatively simple biophysical model with computation and artificial intelligence. A first somewhat surprising fact, shown in Mizraji (1989), is that for this kind of memory the “XOR problem” does not exist. Let us say previously that a dyadic logical operation (such as XOR or Disjunction or Conjunction) requires the definition of a set of truth values$\uptau =\{\mathrm{t},\mathrm{f}\}$, where the “true” value, t, and the “false”, f, are abstract objects. These objects can be represented, respectively, in multiple ways: by letters (T, F), by numbers (1, 0) and also by column vectors of the same dimension (s, n). A dyadic operation like XOR is an application of type

$$\mathrm{XOR}:\uptau \times\uptau \to\uptau$$

where $\times$ represents the Cartesian product. With these bases we see that an X matrix with the memory structure (9), implements the XOR in a straightforward way:

11 $$\mathrm{X}=\mathrm{n}(\mathrm{s}\otimes \mathrm{s}{)}^{\mathrm{T}}+\mathrm{s}(\mathrm{s}\otimes \mathrm{n}{)}^{\mathrm{T}}+\mathrm{s}(\mathrm{n}\otimes \mathrm{s}{)}^{\mathrm{T}}+\mathrm{n}(\mathrm{n}\otimes \mathrm{n}{)}^{\mathrm{T}}$$

Consequently, $\mathrm{X}(\mathrm{s}\otimes \mathrm{s})=\mathrm{X}(\mathrm{n}\otimes \mathrm{n})=\mathrm{n}$ and $\mathrm{X}(\mathrm{s}\otimes \mathrm{n})=\mathrm{X}(\mathrm{n}\otimes \mathrm{s})=\mathrm{s}$, which gives us a matrix–vector version of the XOR operation. This has been extended in multiple directions showing the potential of this formalism to represent a very wide variety of logical operations through matrix memory modules (Mizraji 1992, 2008a; Mizraji and Lin 2011). In Valle-Lisboa et al. (2005), it is shown that through the Widrow-Hoff algorithm, the memory X shown in Eq. (11) is acquired in a minimum number of steps.

Another interesting connection between artificial intelligence and neural models arises from a problem established by Minsky (1988), related to the access to diagnoses from successive partial data. This problem and a possible solution have been analyzed in terms of a recursive process that occurs in a network of neural modules where standard associative memories interact with context-dependent memories (Pomi and Mizraji 2001). A similar neural approach has been used to analyze subtle aspects of performance evolution in the case of medical diagnosis (Pomi and Olivera 2006; Pomi 2017).

Pioneering research on the use of neural models for symptom analysis of mental disorders was published by Hoffman and McGlashan (1997) to explain auditory hallucinations in schizophrenia. This work was inspired by the famous Elman model (Elman 1990), adapted to associate phonetic inputs with conceptual interpretations. Based on these results, Valle-Lisboa et al. (2005) published an investigation that included in the topology of the Elman model a multiplicative contextualization of the conceptual interpretations and the contents of the working memory, obtaining results similar to those found by Hoffman and McGlashan. Let us mention that the original Elman model included hidden layers, however, in the model by Valle-Lisboa et al., pattern multiplication makes hidden layers no longer necessary.

An abundant investigation is carried out today on the structure of semantic spaces. We point out two examples in which the Kronecker product was used to organize these spaces into subspaces selected by multiplicative contexts. In Pomi and Mizraji (2004), a minimal model is shown where the associations between patterns are parameterized by vector contexts. This allows us to divide the space into two layers with independent associations in each of them. However, the entire structure is subtly superimposed on the memory matrix. In Valle-Lisboa et al. (2014) applies the previous idea as a way of representing semantic networks organized by multiplicative contexts. This makes it possible to develop a model of language production that illustrates aspects of its physiological execution and the way in which this execution deteriorates in some mental disorders such as schizophrenia (Valle-Lisboa et al. 2014).

An extension of the contextualized memory model described in Eq. (9) results from introducing multiplicative contexts that are also associated with the outputs (Mizraji 2008b; Mizraji et al. 2009). In these reports, the output is also associated with a multiplicative context, so the tensor structure of this model is (C', O, C, I), where C' is the context associated with output O. The motivation for this contextualization of the output is as follows: In cognitive processes, e.g., thought, neural modules create a “dialogue” with each other, such that the output of a memory M1, for example, is the input of a memory M2. We will assume that the specific access from the output of memory M1 to the memory M2 requires a kind of specific password. That password is the output context.

In this case, the full output is a context-modulated vector with the structure

$${\mathrm{d}}_{\mathrm{ij}}=\mathrm{p}{\mathrm{^{\prime}}}_{\mathrm{i}}\otimes {\mathrm{g}}_{\mathrm{ij}}$$

where $\mathrm{p}{\mathrm{^{\prime}}}_{\mathrm{i}}$ is the context and ${\mathrm{g}}_{\mathrm{ij}}$ is the associated output. A memory module with double contextualization has the following structure

12 $$\mathrm{M}={\sum }_{\mathrm{i},\mathrm{j}}(\mathrm{p}{\mathrm{^{\prime}}}_{\mathrm{i}}\otimes {\mathrm{g}}_{\mathrm{ij}})({\mathrm{p}}_{\mathrm{i}}\otimes {\mathrm{f}}_{\mathrm{ij}}{)}^{\mathrm{T}}$$

Property (d) of the Kronecker product gives us another expression for this memory:

13 $$\mathrm{M}={\sum }_{\mathrm{i},\mathrm{j}}(\mathrm{p}{\mathrm{^{\prime}}}_{\mathrm{i}}{{\mathrm{p}}_{\mathrm{i}}}^{\mathrm{T}}\otimes {\mathrm{g}}_{\mathrm{ij}}{{\mathrm{f}}_{\mathrm{ij}}}^{\mathrm{T}})={\sum }_{\mathrm{i}}\left(\mathrm{p}{\mathrm{^{\prime}}}_{\mathrm{i}}{{\mathrm{p}}_{\mathrm{i}}}^{\mathrm{T}}\otimes {\sum }_{\mathrm{j}}{\mathrm{g}}_{\mathrm{ij}}{{\mathrm{f}}_{\mathrm{ij}}}^{\mathrm{T}}\right)$$

This representation shows that the context pairs generate a (generally spatially distributed) partition of the entire memory module into sub-modules segregated by the context pair. To illustrate this in a formally simple situation, let us imagine that for each term in Eq. (13) the contexts are unit vectors (vectors with a 1 at position i and 0 at all others) with the same dimension n. So, it turns out that

$$\begin{array}{cc}{\mathrm{e}}_{\mathrm{i}}{{\mathrm{e}}_{\mathrm{i}}}^{\mathrm{T}}=[{\updelta }_{\mathrm{ji}}{\updelta }_{\mathrm{ij}}]\in {\mathbb{R}}^{\mathrm{n}\times \mathrm{n}}& \mathrm{i},\mathrm{ j }= 1, \dots ,\mathrm{ n}\end{array}$$

being ${\updelta }_{\mathrm{\alpha \beta }}=\hspace{0.33em}1$ iff $\mathrm{\alpha }=\hspace{0.33em}\upbeta$ and ${\updelta }_{\mathrm{\alpha \beta }}=\hspace{0.33em}0$ iff $\mathrm{\alpha }\ne\upbeta$. Hence, ${\mathrm{e}}_{\mathrm{i}}{{\mathrm{e}}_{\mathrm{i}}}^{\mathrm{T}}={\mathrm{I}}_{\mathrm{n}}$, the identity matrix of order n. The memory is now

$$\mathrm{M}={\sum }_{\mathrm{i}}\left({\mathrm{e}}_{\mathrm{i}}{{\mathrm{e}}_{\mathrm{i}}}^{\mathrm{T}}\otimes {\sum }_{\mathrm{j}}{\mathrm{g}}_{\mathrm{ij}}{{\mathrm{f}}_{\mathrm{ij}}}^{\mathrm{T}}\right)$$

whose explicit structure shows the partition of memory into sub-modules:

$$\mathrm{M}=\left[\begin{array}{cccc}{\sum }_{\mathrm{j}}{\mathrm{g}}_{1\mathrm{j}}{{\mathrm{f}}_{1\mathrm{j}}}^{\mathrm{T}}& 0& \cdots & 0\\ 0& {\sum }_{\mathrm{j}}{\mathrm{g}}_{2\mathrm{j}}{{\mathrm{f}}_{2\mathrm{j}}}^{\mathrm{T}}& \cdots & 0\\ \vdots & \vdots & \ddots & \vdots \\ 0& 0& \cdots & {\sum }_{\mathrm{j}}{\mathrm{g}}_{\mathrm{nj}}{{\mathrm{f}}_{\mathrm{nj}}}^{\mathrm{T}}\end{array}\right]$$

An extension of this idea shows the usefulness of this formalism to analyze a topographic organization of memory modules that generate diverse associative trajectories (Pomi et al. 2018).

Finally, let’s point out that this multiplicative formalism can help to understand how simple words like prepositions (“On”, “Under”, “In”, etc.) encode complicated topological relationships that the brain is capable of computing. An approach to this difficult problem has been published by Mizraji and Lin (2015) based on the computational capabilities of multiplicative contexts. In that article, the authors present a hierarchical model with three neural layers, ranging from concrete natural language phrases to increasingly abstract and general encodings.

Hence, from the point of view of biophysics, the original plan that Warren McCulloch wanted to carry out (McCulloch 1967; Perkel 1988), to develop a calculation of ideas with his neural networks (this plan quickly failed due to the lack of robustness of his circuits) is now achievable with context-dependent associative memories. But artificial intelligence, which emerged along with the early models of neural networks, as noted above, followed a tortuous path, away from neural models, through symbolism and functionalism, only to make a strong comeback in the second decade of this century to neuro-inspired models, with the dizzying development of multilayer models called deep-learning. Although these models are in principle computational artifacts, their evolution has converged to dialogue with the biology and dynamic organization of the cerebral cortex and the complex sensory processing of humans, as will be discussed in the next section. There we will discuss the presence of multiplicative processing in some of the most important models in the area, and the possible reunion of these artificial models with neurobiological computing.

But before that, we want to notice that, interestingly, tensor product nets became recently part of the computational tools of deep learning models (see, for example Yu et al. (2012); Cohen et al. (2016); Huang et al. (2017); Newman et al. (2018)). Eventually this could become another example of natural and cultural convergent evolution of computational solutions.

## Multiplication in deep neural networks

As we mentioned before, artificial neural networks were born as neurocognitive models (McCulloch and Pitts 1943; Rosenblatt 1958; Rumelhart and McClelland 1986) but present-day artificial neural networks (ANNs) involve several non-biological procedures that render them unrealistic as models of neuronal computation. Among them, the backpropagation algorithm — in its many instantiations — remains a doubtful procedure in the brain (but see Grüning 2007). In fact, this has prompted many of the most influential researchers in ANNs to look for other methods that can accomplish a similar computation (Lillicrap et al. 2020). There is another strand of computational models of cognition involving Bayesian learning (Yang and Piantadosi, 2022), that are even further removed from neurobiology, as they are formulated at the algorithmic level. Although multiplicative processes can be used to re-implement these models as neural networks (Cabana et al. 2016) we will not review them here.

Nevertheless, the lack of reality of current models only affects the training stage of the network. It might be the case that the computations performed by the networks are close to the actual neurobiological computations, and only depart from biology when the parameters need to be learned. There is some evidence that this is the case, as there is a growing body of research, for instance in visual processing using convolutional neural networks (Yamins and DiCarlo 2016) and also in language (Caucheteux and King 2020, 2022; Schrimpf et al. 2021) and speech processing (Millet et al. 2022) using transformer networks, that finds a strong correlation between brain areas and network layers. Moreover, in all of these examples, the higher the correlations between model layers and brain layers, the better the models perform in comparison with humans (see for instance, Fig. 6 in Schrimpf et al 2021).

Thus, at least part of the computations performed by artificial neural networks uses similar internal representations and similar computations as biological networks. In many cases, these computations involve the multiplication of activity. We stress here that whereas matrix–vector multiplications are conceptualized as a simple way to implement synaptic weights, activity multiplications require the particular cellular biophysics properties we are arguing for in this review. Another point to notice is that ANNs used in Artificial Intelligence are based on tensor representations. For instance, notice that Tensor Flow, one of the first Python libraries created to train deep networks, use intensively tensor-based computations. Thus, from the beginning, the use of tensor algebra is natural in these models. But we will show that the connection runs deeper than this.

Multiplication of activity is explicitly used in Long-Short Term Memories (LSTMs) for gating (Hochreiter and Schmidhuber 1997). It is also used, though this is implicit in the equations, in transformer networks and many attention-based networks (Vaswani et al. 2017). It is not explicitly used in most convolutional neural networks or autoencoders, the other popular neural network models. In the following, we take a closer look at the role of multiplication in two of the most important Artificial Neural Network models used in Artificial Intelligence, namely, transformers and LSTMs.

### LSTMs, the unstable gradient problem, and multiplication

Recurrent neural networks (RNN) have been influential models since their creation (Elman 1990; Pollack 1990). Their main feature is that they include a form of transient memory that after training represents important abstract features of the problem they are set to solve, and this in turn reflects the connection (synaptic) weights the network learned. Thus, the early simple recurrent networks were able to discover the presence of different word categories in a linguistic input generated with a simplified grammar, without any grammatical preprogramming (Elman 1990). Crucial for this performance was that they were trained to predict the next word in their input, a task that continues to be central in present-day Large Language Models (LLMs). The early models like that of Elman used a simple backpropagation algorithm, and later backpropagation through time (Williams and Zipser 1989). A well-known property of natural language that these networks tried to model, is long-distance dependencies, i.e., the dependency of the processing of some words on previous words, that are at an arbitrary distance from the current word (Chomsky 1956).

Although these models could potentially accommodate any dependency between words, they suffered from several drawbacks that made them unsuitable for scaling up. In particular, they suffered from the exploding gradient problem or vanishing gradient problem (Hochreiter 1998). In deep networks and recurrent networks, there is the possibility that the error signal that backpropagates either vanishes when going back several time steps (or network layers) or grows without limits. In any case, this makes the learning of long dependencies in recurrent networks or training the deepest layers in deep networks, quite hard. Hochreiter and Schmidhuber introduced Long Short-Term Memories to propose a solution to the vanishing gradient problem (Hochreiter and Schmidhuber 1997).

In the analysis performed by Hochreiter (see also Bengio et al. 1993), it is shown that the output of a hidden unit should be a linear function of its input if the gradient is not to explode or vanish; if this is the case, its derivative is constant, and an appropriate weight can make the transmission of error to be backpropagated without change. This creates other problems though, as the unit would transmit the errors to units that should not change and be influenced by other inputs that should not affect it.

**Fig. 3:**
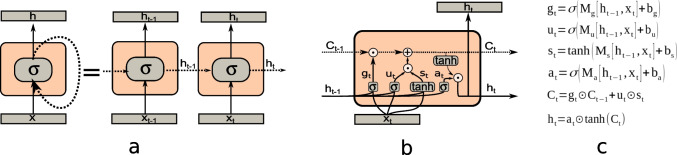
Recurrent neural networks and their unfolded representation. **a** A simple recurrent network (left) and its unfolded version (right). Each time step a vector representing a word (x_t_) enters the network; in the hidden layer, the input and context vector (h_t_) are multiplied by a matrix and then they are non-linearly transformed by the function σ, classically a logistic function. This output is the next context vector and is further processed by superior layers (not shown) to produce the output. **b** A LSTM block as part of an unfolded LSTM. In each time-step, each block receives the input vector x_t_, the previous context vector (h_t-1_) and the previous “cell-state” vector (C_t-1_) and then it outputs a new cell state and a new context. In each time the context, input and cell state are processed through different matrices and functions. **c** Detailed equations showing the information flow within a LSTM block. In all cases σ refers to a squashing function, in general a logistic function. The vectors in square brackets [h,x] denote the concatenation of h and x vectors. The symbol ⨀ is the pointwise vector multiplication. The function tanh is the hyperbolic tangent

Multiplicative gating enters here as a way to solve the vanishing gradient problem without affecting the effect of other inputs. Although the details of the LSTMs are outside the scope of this review, let us describe briefly how multiplication enters in this architecture. The idea is to have context units that keep a value during an arbitrary number of time-steps (or words in the sequence) and use multiplicative gates to control the input, output, and change of each of the cell states. Each layer thus consists of the activity of a set of “cell states” and “hidden states”. During each time step, both state vectors (the cell and context vectors) are passed to the following time step. The input vector x_t_ and the hidden vectors h_t_ are transformed by a layer that outputs a vector of activities g_t_ (Fig. 3b, c). Usually, a logistic activation function is used, implying that the components of this vector belong to the interval [0,1). The first multiplicative gate performs the point-wise multiplication of this vector to the cell state vector. It is interpreted that this gate can “erase” those components of the cell state that should be turned off according to the current input and the previous context. The input and hidden state are also processed by two other layers, one using a logistic activation function, producing u_t_, and another using a hyperbolic tangent activation function, producing s_t_. The output of these two layers is pointwise multiplied by the second multiplicative gate. The role of the multiplication is to select those components in the interval (-1,1) that should be added to the cell state vector that will pass to the other time step. The result of this multiplication is added to the cell state (that has already been multiplied by the first gate), effectively storing a new cell state. The final gate uses the hyperbolic tangent of the new cell state and the output of another layer that processes the state and input producing a_t_, to update the hidden state. The hidden state is broadcasted to the upper output layers. Thus, there are four matrices that need to be learned and three pointwise vector multiplication operations (see Fig. 3b and c for the details and Olah 2015). We emphasize here that these pointwise multiplications, by no means imply a localist representation, as they are based on vectors, and thus admit distributed representations; localist representations are a special case (using sparse vectors).

As this resumed explanation shows, multiplication is thus used in LSTMs, its main purpose is solving the vanishing gradient problem, which specifically applies to learning with backpropagation, a biologically unrealistic learning algorithm. Nevertheless, it has other desirable properties. In particular, the reason why it solves the vanishing gradient problem is that the network learns to store, erase and use different cell-states according to context and past experience. This implies a form of controlling the flux of information in and out of transient storage. Multiplication is essential to gate information in or out of this storage, and in particular, to erase the information that is irrelevant for a particular context. In this sense, it works in a similar fashion as it does for our context-dependent memory models and it is related to the classical models of Grossberg and coworkers (Carpenter and Grossberg 1981).

When learning highly nonlinear mappings, filtering out irrelevant information is essential, as is explicitly mentioned in early models of (computational) attention, a further level of filtering that was inspired in cognitive attention (Mnih et al. 2014; Petersen and Posner 2012). Without much regard for the precise neurobiological properties, these attention algorithms have been used in machine translation tasks with sequence-to-sequence (seq2seq) models (Bahdanau et al. 2016; Luong et al. 2015). Attention in artificial neural networks denotes a set of modules or procedures that enhance filtering, and they are in particular crucial for the working of one of the models that is revolutionizing all areas of artificial intelligence; the transformer. We present how attention works together with the transformer architecture in the following section.

### Transformers and attention

**Fig. 4:**
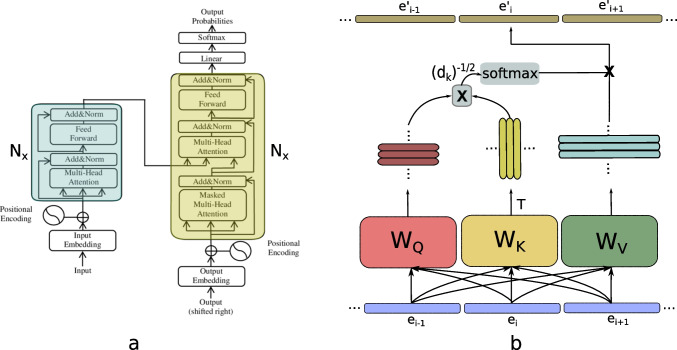
Transformer and multi-head attention architectures. **a** Transformer architecture (Vaswani et al., 2017; Jia, 2019). The left structure is called the encoder, the right network the decoder, and many of these network blocks can be concatenated. In the figure, only the Nx block is shown. **b** The inner workings of one of the heads in multi-head attention. In each head, each input vector (called embedding as it can be an internal vector) is multiplied in parallel by three matrices; a matrix W_Q_ that produces query vectors, a matrix W_K_ that produces key vectors and a matrix W_v_ that produces value vectors. Each input embedding e_i_ is transformed into a weighted sum of all value vectors into e_i_’ (see text). The output of all heads is then concatenated and multiplied by an output matrix before entering the next layer

One of the most important innovations in neural networks during the last few years is the Transformer architecture (Fig. 4a). Although several types of transformer architectures are available, the ones used for machine translation are particularly common, and are the basis for the well-known Large Language Models like the family of models GPT-x (Brown et al. 2020), which are the basis for popular applications such as ChatGPT, and are nowadays the subject of an intense research activity (Liu et al. 2023). A description of the details of the transformer architecture and reasons for each architectural decision are outside the scope of this review. The reader should consult recent reviews about the topic (Ghojogh and Ghodsi 2020).

What is important for our review is the presence of attention mechanisms. Transformers include attention mechanisms as a way to solve long-term dependency problems. The first artificial neural networks that used attention were applied to vision (Mnih et al. 2014) and machine translation (Bahdanau et al. 2016). The machine translation models were initially based on Long Short-Term Memories. Several types of attention were devised (see below), but the most conspicuous type nowadays is the *multi-head attention*, which is the type used by transformers. The main idea behind all attention mechanisms proposed is that these mechanisms provide dynamically varying weights that can filter out the context that is irrelevant for processing the current input, while allowing this processing to be influenced by potentially distant context. As an example, a seq2seq model is a neural network that can take an input sequence in one language and output the corresponding sequence in another language. The initial seq2seq models used two LSTMs, one called the encoder, the other the decoder. The encoder receives a word one at a time, and outputs a context vector **h**_t_ after receiving t words. The context vector is used as an initial context vector in the decoder, that receives also as input a vector coding for the beginning of a sentence. The decoder computes the new context vector and outputs a word. The process continues until the decoder outputs a termination word. Using a single **h**_t_ vector to encode the whole input sequence has several limitations. Ideally all the context vectors produced during the processing of the input sequence have information about its meaning. But not all the context vectors are equally relevant for the meaning of each particular output. This is where attention is relevant. By learning the weights associated with attention, the network learns to select only the relevant context to process each input.

There are several dimensions to consider in order to classify the different types of attention (Chaudhari et al. 2021). For instance, it is relevant to consider whether the mechanism is global or local (Luong et al. 2015), whether is uses a hard attention (Xu et al. 2016) or soft attention (Bahdanau et al. 2016), and whether attention is applied at one or several levels (Yang et al. 2016) among other relevant things. In all cases, attention involves the multiplication of vectors of activities and vectors of weights derived from activities, which is the type of multiplication that we argue is central for effective cognitive computation. In the following, we demonstrate this by concentrating in multihead attention as an example.

### Multi-head attention

In the paper titled “Attention is all you need”, Vaswani and collaborators (Vaswani et al. 2017) proposed the use of multi-head attention as a form both of self-attention and encoder-decoder attention. In Fig. 4 b, we present the general architecture of the multi-head attention mechanism.

Multihead attention refers to the presence of several parallel channels that implement complementary attention functions. Each head includes three matrices that are learned during model training. Each of the attention heads processes all word embeddings by these three matrices, transforming each embedding into a query, a key and a value vector. The dot product of each query and each key is then scaled by the square root of the key dimensions and submitted to softmax. Softmax is a function that takes all the component activities of a vector and produces a new vector of probabilities, i.e., the sum of all components is 1 and each component is proportional to the exponential function of the activity. The result is used to weight the value vectors. In matrix form, if (following Alammar 2018) E is a matrix whose rows are the embeddings coming from the previous layer, then we define,

$$\mathrm{Q}={\mathrm{EW}}_{\mathrm{Q}}$$

$$\mathrm{K}={\mathrm{EW}}_{\mathrm{K}}$$

$$\mathrm{V}={\mathrm{EW}}_{\mathrm{V}}$$

where Q is called the queries matrix, K, the keywords matrix and V the values matrix. Then each attention head produces an output:

$$\mathrm{Z}=\mathrm{softmax}\left(\frac{{\mathrm{QK}}^{\mathrm{T}}}{\sqrt{{\mathrm{d}}_{\mathrm{k}}}}\right)\mathrm{V}$$

The rows of Z are further processed by the other layers in the case of the encoder self-attention part of the transformer. In the case of encoder-decoder attention, the output of the encoder are keys and values that are used together with decoder queries and the current input to predict the next word in the decoder.

In order to see why these operations are important for our review, consider the individual components of the output. The outputs of each self-attention head for each word embedding (z_*i*_) are,

$${\mathrm{z}}_{\mathrm{i}}={\sum }_{\mathrm{j}=1}^{\mathrm{n}}{\mathrm{v}}_{\mathrm{j}}\mathrm{softmax}(\frac{{\sum }_{\mathrm{l}=1}^{{\mathrm{d}}_{\mathrm{k}}}{\mathrm{q}}_{\mathrm{i}}(\mathrm{l}){\mathrm{k}}_{\mathrm{j}}(\mathrm{l})}{\sqrt{{\mathrm{d}}_{\mathrm{k}}}})$$

with v_j_ the value vector associated with the input embedding x_j_, q_i_(l) the l-th component of the i-th query vector, k_j_(l) the l-th component of the j-th keyword vector.

The *z* vectors are a linear combination of value vectors v, but the coefficients are themselves calculated from the embeddings by applying softmax on top of the (scaled) dot product of all query and value vectors. This implies that this linear combination already includes a multiplication of activities. There is also a multiplication of activity in the dot product of the query and key vectors. These multiplications are essential. The dot product between q and k vectors filters words according to their relative importance (given the context). The importance is used to weight each value vector by multiplication. In this sense this is reminiscent of the double filtering process our tensor model is based on, a connection that we are currently exploring.

Although the way attention is usually presented is not exactly an input–output feedforward network nor as a recurrent network, it can be made to comply these biologically related architectures. In this sense, the important aspect of these models is that they show the paramount relevance of multiplication in state-of-the-art models.

## Conclusions and perspectives

Multiplication greatly enhances the capabilities of neural models, and it is included in several classical models of cognitive processing like tensor matrix memories (Mizraji 1989), tensor models of symbolic processing (Smolensky 1990), or pattern recognition machines (like functional-link nets, Pao 1989). It is also used in state-of-the-art Artificial Intelligence tools, like Long Short-Term Memories (LSTM, Hochreiter and Schmidhuber 1997) and Transformers (Vaswani et al 2017). Newer models based on Structured Space Models (Dao et al. 2022) also include forms of multiplication. The growing importance of this basic operation opens two questions.

On one side, multiplications allow for the flexible modulation of input–output mappings, which in turn permits neural networks to implement *gratuitous* mappings, i.e., computations that are not dependent on the details of the input (much in the way an allosteric modulator allows for the regulation of a metabolic pathway by chemical compounds unrelated to the pathway, as proposed by Monod 1967). Is the presence of gratuitous interactions an inescapable design feature of intelligent systems? If the answer is positive, in this sense classical multiplicative neural networks, but also state-of-the art intelligent machines realize the postulate that Ross Ashby proposed as necessary for a system to present adaptive behaviors (Ashby 1956, 1960; Mizraji and Lin 2011). There is an opportunity then, to understand theoretically what is required for this type of intelligent computation. Multiplication will likely be part of the necessary ingredients of this understanding.

This leads to the other question. What are the concrete material bases for multiplying signals in the nervous system? Most simple expositions of synaptic integration start with an additive, linear summation model, not different from the usual connectionist information processing unit (Kandel 2001; Rumelhart, Hinton and McClelland 1986). It is clear that dendritic trees, shunting inhibition, des-inhibition, and nonlinear receptor dynamics drastically modify this simplistic picture. What precise combination of these and other ingredients are actually used by different parts of the nervous system? An interdisciplinary effort starting with computational models and ending at the molecular level to explain these aspects is needed to unravel the secrets that synapses and circuitry hide and what it takes to be intelligent.

## Data Availability

As this manuscript is a review, we do not use any new unpublished data.
